# Interactome Analysis of Microtubule-targeting Agents Reveals Cytotoxicity Bases in Normal Cells

**DOI:** 10.1016/j.gpb.2017.04.006

**Published:** 2017-12-12

**Authors:** Andrés Julián Gutiérrez-Escobar, Gina Méndez-Callejas

**Affiliations:** Grupo de Investigaciones Biomédicas y Genética Aplicada – GIBGA, Universidad de Ciencias Aplicadas y Ambientales U.D.C.A., Bogotá 111166, Colombia

**Keywords:** Cancer treatment, Microtubule-targeting agent, Interactome analysis, Cancer biology, Apoptosis

## Abstract

Cancer causes millions of deaths annually and **microtubule-targeting agents** (MTAs) are the most commonly-used anti-cancer drugs. However, the high toxicity of MTAs on normal cells raises great concern. Due to the non-selectivity of MTA targets, we analyzed the interaction network in a non-cancerous human cell. Subnetworks of fourteen MTAs were reconstructed and the merged network was compared against a randomized network to evaluate the functional richness. We found that 71.4% of the MTA interactome nodes are shared, which affects cellular processes such as **apoptosis**, cell differentiation, cell cycle control, stress response, and regulation of energy metabolism. Additionally, possible secondary targets were identified as client proteins of interphase microtubules. MTAs affect apoptosis signaling pathways by interacting with client proteins of interphase microtubules, suggesting that their primary targets are non-tumor cells. The paclitaxel and doxorubicin networks share essential topological axes, suggesting synergistic effects. This may explain the exacerbated toxicity observed when paclitaxel and doxorubicin are used in combination for **cancer treatment**.

## Introduction

In 2013, 14.9 million cases of cancer, 8.2 million deaths, and 196.3 million disability adjusted life years (DALYs) were reported [1]. The standardized incidence levels by age for all cancer types combined increased by more than 10% in 113 countries from 1990 to 2013 [1], with developing countries among the most affected. For this reason, the development of effective cancer drugs is a global priority.

Microtubules are among the most extensively studied therapeutic targets in cancer treatment [2], [3]. Made up of α and β tubulin protofilaments, microtubules can serve as the basis of a cellular network, which are essential for determining cell shape and organization through the movement of organelles, such as the nucleus, mitochondria, endoplasmic reticulum, and Golgi apparatus [4], [5], [6]. In addition, this network plays a regulatory role in cell migration and adhesion as well [6], [7].

Microtubule-targeting agents (MTAs) can affect microtubule stability, leading to disruption of the mitotic spindle and cell death [8], and are therefore one of the most effective classes of drugs used in chemotherapy against cancer. According to their binding property with tubulin, MTAs are classified as stabilizing agents (known as vinca alkaloids) [9], [10], which bind the tubulin polymer, and destabilizers (known as taxanes) [11], [12], [13], which bind tubulin dimers. The stabilizing vinca-type drugs, including vincristine, vinblastine, and vinorelbine, are used for haematological cancers such as lymphomas and leukaemia, whereas destabilizing taxane-type drugs, including paclitaxel and docetaxel, are used to treat solid cancers such as breast, ovary, and oesophageal cancer [14].

However, high *in vivo* cytotoxicity and increased tumor resistance have been observed with the usage of MTAs [15], [16], since they can recognize microtubules of interphase cells [17]. MTAs kill rapidly-dividing cells by arresting them in mitosis [18], but how MTAs kill slowly-dividing tumor cells has not been fully described. It has been proposed that MTAs interfere with microtubule trafficking system in prostate cancer patients [19], enabling the use of MTAs in a combination regimen with DNA-damaging agents (DDAs), such as doxorubicin, for cancer treatment [20].

One method for studying the interactions between drugs and the human proteome is to use network theory, in which proteins and/or chemical compounds are represented by nodes, and the interactions between them are represented by edges. As a result, inference of cellular processes affected by certain drugs can be inferred by studying the connections between nodes. Protein network reconstruction has allowed a better understanding of the physiopathological mechanisms of cancer, identified genes associated with specific pathologies to improve disease classification, and defined specific cancer sub-networks important for the identification of therapeutic targets [21], [22], [23], [24], [25].

In this study, we created an integrated interaction network between MTAs and the proteome of a non-cancerous cell to identify the essential topological axes of its functional profile. Potential cellular mechanisms associated with the cytotoxicity of MTAs are also described.

## Results

### Different biological function detected from MTA subnetworks

The related data for MTA subnetworks (Table S1) showed that the microtubule inhibitor 2-methoxyestradiol triggers the metabolism of flavonoids, lipids, vitamins, and podophyllotoxins. In addition, association of 2-methoxyestradiol with cellular adhesion and adiponectin secretion was also detected. Colchicine and vincristine were involved in the cellular stress, death, and proliferation as well. Besides, combretastatin A4, epothilone, epothilone B, and vinblastine are shown to induce the formation of protein complexes and microtubule-based organelle movements, and might induce apoptosis of lymphocytes. Spongistatin was involved in the regulation of NF-κβ, whereas vindesine, vinorelbine, and tasidotin induce cytotoxicity. Finally, nocodazole, noscapine, and paclitaxel were involved in protein phosphorylation, nuclear membrane reorganization, and apoptosis.

### Integrated MTA interactome shows a common proteome core

**Figure 1:**
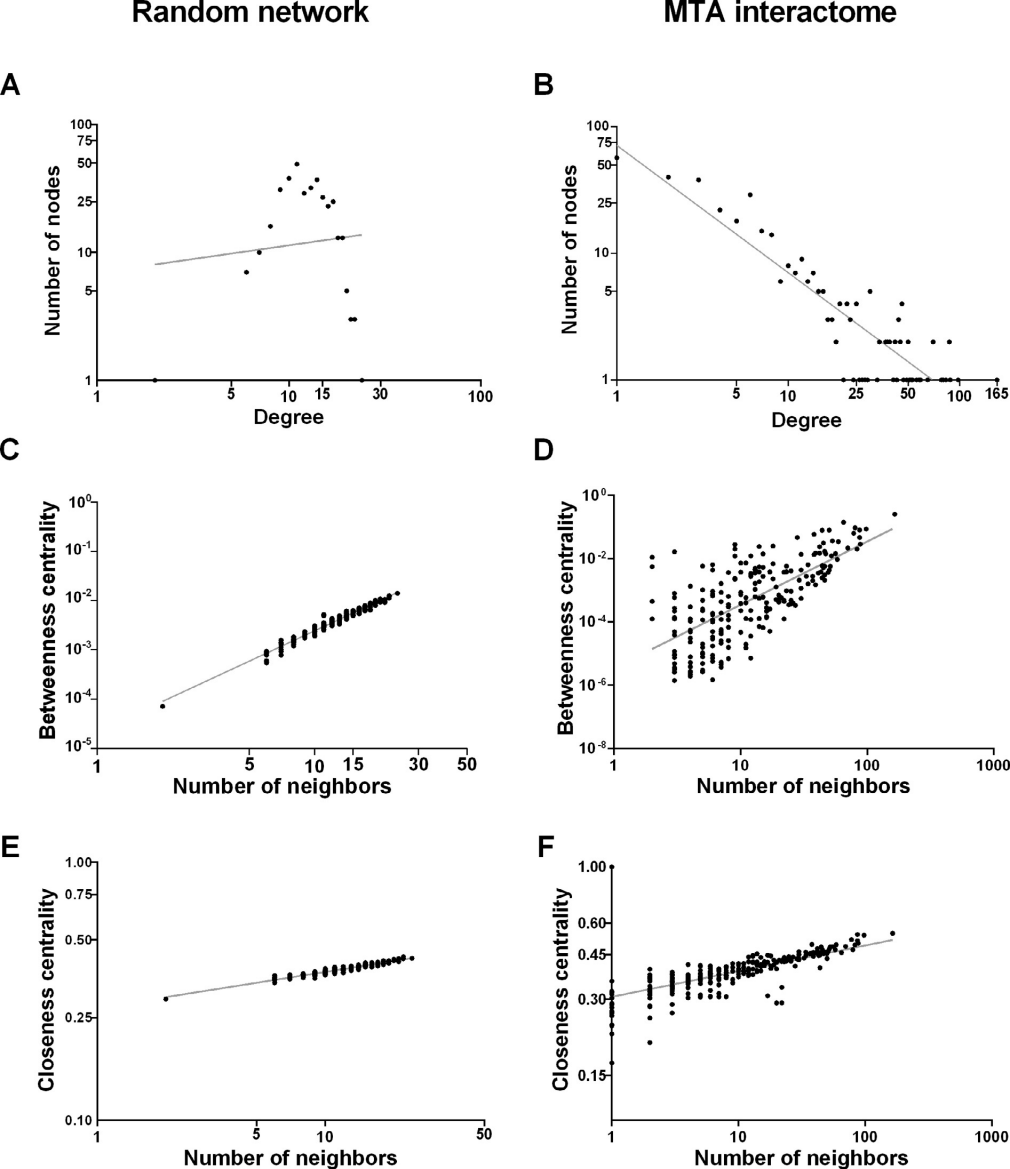
**Comparative analysis of the topological properties between the random network and the MTA interactome** **A.** Node distribution according to the node degree relative to the number of neighbours for random network. **B.** Node distribution according to the node degree relative to the number of neighbours for MTA interactome. **C.** Node distribution for betweenness centrality relative to neighbour distribution for random network. **D.** Node distribution for betweenness centrality relative to neighbour distribution for MTA interactome. **E.** Node distribution for closeness centrality relative to the number of neighbours for random network. **F.** Node distribution for closeness centrality relative to the number of neighbours for MTA interactome. MTA, microtubule-targeting agent.

In order to establish the common interactome for the MTAs, we merged all the subnetworks for MTAs together. It was determined that 71.4% of the integrated network shared a common core formed by 363 nodes and 2327 connections (Figure 1).

To create a comparison pattern for the integrated network, a randomized network was generated with similar topological characteristics. We observed that the integrated network had a positive correlation between the number of nodes and the node degree (*R* = 0.935; *R*^2^ = 0.799) compared with the random network (*R* =  −0.045; *R*^2^ = 0.04; Figure 1A and B). Additionally, the distribution of betweenness centrality (BC) and closeness centrality (CC) indicated that the integrated network was more compact than the random network (Figure 1C−F). The mean value for the clustering coefficient for the integrated network (0.583) was significantly higher than that for the random network (0.035) (Wilcoxon *Z*-value = −12.2321, *P* = 1E−5, significant at *P* < 0.01). Likewise, the mean *k* value for the integrated network (26.122) was significantly higher than that for the random network (12.822) (Wilcoxon *Z*-value = −12.322, *P* = 1E−5, significant at *P* < 0.01). These results suggest that all nodes in the integrated network have a distribution that could be attributed to their functional properties, indicating the presence of functionally-essential nodes within the network (topological axes).

To identify the topological axes in the integrated network, the distribution of the networks according to the node degree was analyzed. This distribution showed that the different tubulin conformations were the most connected nodes. Moreover, there are also other topological axes, including *TP53*, *AKT1*, *CDK1*, *PCCNA*, *JUN*, *VEGFα*, *CASP3*, *CCNB1*, and *RAC1*. The encoded proteins regulate cellular processes such as apoptosis, cell differentiation, cell cycle control, replication control in eukaryotes, stress response, and energy metabolism regulation. In addition, we also observed high degree values for paclitaxel and docetaxel, indicating that these MTAs can make many connections with the human proteome (Table 1).

**Table 1:** Top 50 essential nodes of the integrated MTA network

**Essential nodes**	**Degree**	**Betweenness centrality**	**Closeness centrality**	**Stress**
*TUBB2A*	268	0.126	0.558	612,662
*TUBB4B*	212	0.020	0.487	255,818
*TUBB2B*	210	0.050	0.521	343,900
*TUBB4A*	203	0.017	0.483	233,252
*UBC*	198	0.032	0.489	264,296
*TUBA1A*	191	0.040	0.500	298,950
*TUBB1*	181	0.053	0.500	261,470
*TUBA4A*	180	0.012	0.474	165,748
*TUBB6*	177	0.009	0.455	124,806
*TUBA1C*	168	0.011	0.476	174,648
*TUBA1B*	165	0.010	0.468	142,518
Paclitaxel	165	0.141	0.488	1,184,898
*TP53*	150	0.078	0.531	603,234
*AKT1*	139	0.075	0.522	786,472
*TUBA3D*	133	0.005	0.456	91,826
*CDK1*	124	0.039	0.514	450,050
*TUBB8*	121	0.004	0.434	67,272
*HSP90AA1*	115	0.005	0.458	76,740
*CDK2*	111	0.030	0.508	529,562
*TUBG1*	106	0.007	0.444	69,732
*TUBA3E*	102	0.001	0.425	29,842
*TUBA3C*	100	0.001	0.426	28,990
*PCNA*	100	0.031	0.498	382,750
*JUN*	98	0.031	0.479	180,766
*TUBA8*	98	9.73E−04	0.426	23,082
*ACTB*	90	0.006	0.449	82,236
*HSP90AB1*	89	0.003	0.445	52,784
*TUBAL3*	88	7.64E−04	0.421	21,772
*DYNC1H1*	88	8.44E−04	0.422	17,428
*VEGFA*	87	0.024	0.458	286,248
*CASP3*	87	0.039	0.484	406,936
*CCNB1*	87	0.015	0.487	286,352
*DNAH8*	85	0.002	0.445	51,444
*POTEF*	84	0.003	0.446	61,686
*RAC1*	81	0.020	0.468	1961,84
Docetaxel	80	0.055	0.485	273,726
*KIF11*	79	6.95E−04	0.421	13,264
*GAPDH*	79	0.0030	0.445	49,924
*PTGS2*	78	0.0293	0.460	264,080
*POTEI*	74	0.0012	0.421	22,752
*DYNC2H1*	74	2.40E−04	0.403	4178
*MAPRE1*	73	0.013	0.458	148,942
*HDAC9*	73	0.019	0.485	334,388
*PLK1*	73	0.009	0.441	53,276
*POTEJ*	72	0.001	0.421	21,130
*POTEE*	72	0.001	0.421	21,130
*ACTA1*	72	0.003	0.441	45,090
*DNAH9*	71	1.95E−04	0.402	3788
*DNAH17*	71	1.95E−04	0.402	3788

**Figure 2:**
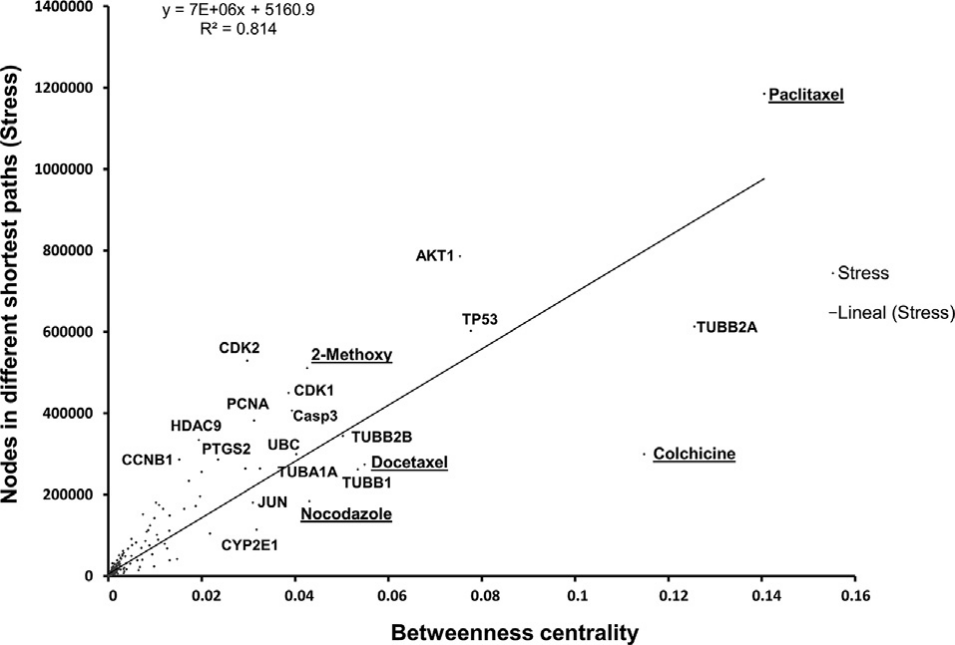
**Essential nodes for information flow in the integrated MTA network** A fitted line and its slope were calculated to identify the most significant nodes and MTAs that modulate the information flow in the integrated network, using the betweenness centrality and stress values. Betweenness centrality represents the number of times a node is visited and stress indicates how many times a particular node is part of different shortest paths. The most relevant MTAs in the information flow of the network are underlined.

To determine the role of these topological axes in information flow, we analyzed the network topology in terms of stress and BC. We found a positive correlation between these two parameters in the integrated network (*R* = 0.897; *R*^2^ = 0.814) (Figure 2). The nodes with the highest stress and BC coincided with those identified using node degree. These results suggest that paclitaxel, *TUBB2A*, *TP53*, and *AKT1* are the topological axes that control and regulate the information flow in the integrated network.

We then performed the cluster analysis of the network to identify the functional profile of its main subnetworks. Consequently, two functional domains were revealed. The first functional domain was related to apoptosis regulation, proliferation, locomotion, and cell migration, whereas the second domain was related to drug catabolism (Table 2). To identify the role of these functional domains in cancer metabolism, we mapped the nodes into the KEGG database. As shown in Table 3, nodes are mapped to pathways related to apoptosis, cell proliferation, and cancer metabolism.

**Table 2:** Functional domains and the associated essential nodes of the integrated MTA network

**Functional domain**	**Cellular processes**	***P* value**	**Adjusted *P* value**	**Essential nodes**
1	Regulation of apoptosis	1.11E−33	2.36E−26	*CASP3*, *CASP8*, *TP53*, paclitaxel, *JUN*, *AKT1*, *PARP1*, *CYCS*, *BAX*, *BCL2*, *FASLG*, *STAT3*, *MAPK1*, *XIAP*, *BIRC5*, *BCL2L11*, *VEGFA*, *CDKN1A*, *PTEN*, *BCL2L1*

	Regulation of cell proliferation	1.61E−23	1.23E−06	*AR*, *CDK2*, *CDK1*, *MDM2*, *PTGS2*, *HSPA4*, *BRCA1*, *GADD45A*, *MAPK14*, docetaxel, *HDAC9*, *CCNB1*, *RB1*, *CTNNB1*, *ABCB1*, *CCL2*

	Protein polymerization	1.98E−11	1.31E−06	*TUBA1A*, *TUBB2B*, epothilones, *TUBA1B*

	Regulation of locomotion	7.26E−14	1.58E−05	*IL8*, *MMP2*, *SERPINE1*, *HBEGF*, *IFNG*, *MMP9*, *CXCR4*

	Negative regulation of leukocyte proliferation	5.72E−03	6.21E−03	*FOS*, *GAL*, *NTS*, *VIP*, *IL2RA*, *MAPK9*

2	Exogenous drug catabolic process	2.93E−11	6.67E−10	*CYP2C19*, *NR1I2*, *CYP1A2*, *CYP2C9*, *ABCC2*, *GSTM5*, *GSTP1*, *ABCB11*, *ABCG2*

**Table 3:** Essential nodes grouped according to their KEGG biological function

**Function**	**Pathway ID**	**Pathway name**	***P* value**	**Essential nodes**
Apoptosis	5200	Pathways in cancer	1.8E−39	*AKT1*, *BAX*, *BCL2*, *BCL2L1*, *BIRC2*, *BIRC5*, *CASP3*, *CASP8*, *CASP9*, *CDH1*, *CDKN1A*, *CYCS*, *EGFR*, *ERBB2*, *FADD*, *FASLG*, *JUN*, *MAPK1*, *MAPK8*, *PIK3CA*, *PTEN*, *STAT3*, *TP53*, *VEGFA*, *XIAP*

	4151	PI3K–Akt signaling pathway	5.94E−15	*AKT1*, *BCL2*, *BCL2L1*, *BCL2L11*, *CASP9*, *CDKN1A*, *EGFR*, *FASLG*, *MAPK1*, *PIK3CA*, *PTEN*, *TP53*, *VEGFA*

	5210	Colorectal cancer	2.28E−22	*AKT1*, *BAX*, *BCL2*, *BIRC5*, *CASP3*, *CASP9*, *CYCS*, *JUN*, *MAPK1*, *MAPK8*, *PIK3CA*, *TP53*

	5206	MicroRNAs in cancer	1.63E−17	*BCL2*, *BCL2L11*, *CASP3*, *CDKN1A*, *EGFR*, *ERBB2*, *MAPK1*, *PIK3CA*, *PTEN*, *STAT3*, *TP53*, *VEGFA*

	5212	Pancreatic cancer	1.01E−19	*AKT1*, *BCL2L1*, *CASP9*, *EGFR*, *ERBB2*, *MAPK1*, *MAPK8*, *PIK3CA*, *STAT3*, *TP53*, *VEGFA*

	4068	FoxO signaling pathway	2.01E−16	*AKT1*, *BCL2L11*, *CDKN1A*, *EGFR*, *FASLG*, *MAPK1*, *MAPK8*, *PIK3CA*, *PTEN*, *SOD2*, *STAT3*

	5205	Proteoglycans in cancer	9.18E−14	*AKT1*, *CASP3*, *CDKN1A*, *EGFR*, *ERBB2*, *FASLG*, *MAPK1*, *PIK3CA*, *STAT3*, *TP53*, *VEGFA*

	5222	Small cell lung cancer	4.22E−16	*AKT1*, *BCL2*, *BCL2L1*, *BIRC2*, *CASP9*, *CYCS*, *PIK3CA*, *PTEN*, *TP53*, *XIAP*

	5215	Prostate cancer	4.35E−16	*AKT1*, *BCL2*, *CASP9*, *CDKN1A*, *EGFR*, *ERBB2*, *MAPK1*, *PIK3CA*, *PTEN*, *TP53*

Proliferation	4110	Cell cycle	0.000145	*CCNB1*, *GADD45A*, *MDM2*, *RB1*

	4115	p53 signaling pathway	0.000679	*CCNB1*, *GADD45A*, *MDM2*

	4668	TNF signaling pathway	0.00241	*CCL2*, *MAPK14*, *PTGS2*

Locomotion	5219	Bladder cancer	5.44E−05	*IL8*, *MMP2*, *MMP9*

	4915	Estrogen signaling pathway	0.000331	*HBEGF*, *MMP2*, *MMP9*

	4670	Leukocyte transendothelial migration	0.000406	*CXCR4*, *MMP2*, *MMP9*

	5205	Proteoglycans in cancer	0.00232	*HBEGF*, *MMP2*, *MMP9*

Metabolism	140	Steroid hormone biosynthesis	4.53E−13	*COMT*, *CYP1A1*, *HSD17B2*, *UGT2B10*, *UGT2B11*, *UGT2B7*

	5204	Chemical carcinogenesis	1.31E−09	*CYP1A1*, *SULT1A1*, *UGT2B10*, *UGT2B11*, *UGT2B7*

	980	Metabolism of xenobiotics by cytochrome P450	2.62E−07	*CYP1A1*, *UGT2B10*, *UGT2B11*, *UGT2B7*

	53	Ascorbate and aldarate metabolism	3.23E−06	*UGT2B10*, *UGT2B11*, *UGT2B7*

	40	Pentose and glucuronate interconversions	6.37E−06	*UGT2B10*, *UGT2B11*, *UGT2B7*

	1100	Metabolic pathways	9.02E−06	*COMT*, *CYP1A1*, *HSD17B2*, *UGT2B10*, *UGT2B11*, *UGT2B7*

	860	Porphyrin and chlorophyll metabolism	9.33E−06	*UGT2B10*, *UGT2B11*, *UGT2B7*

	500	Starch and sucrose metabolism	1.37E−05	*UGT2B10*, *UGT2B11*, *UGT2B7*

Drug catabolism	982	Drug metabolism–cytochrome P450	7.80E−09	*CYP1A2*, *CYP2C19*, *CYP2C9*, *GSTM5*, *GSTP1*

	591	Linoleic acid metabolism	1.38E−05	*CYP1A2*, *CYP2C19*, *CYP2C9*

	2010	ABC transporters	3.31E−05	*ABCB11*, *ABCC2*, *ABCG2*

### The MTA–DDA merged interactome reveals common topological axes

**Figure 3:**
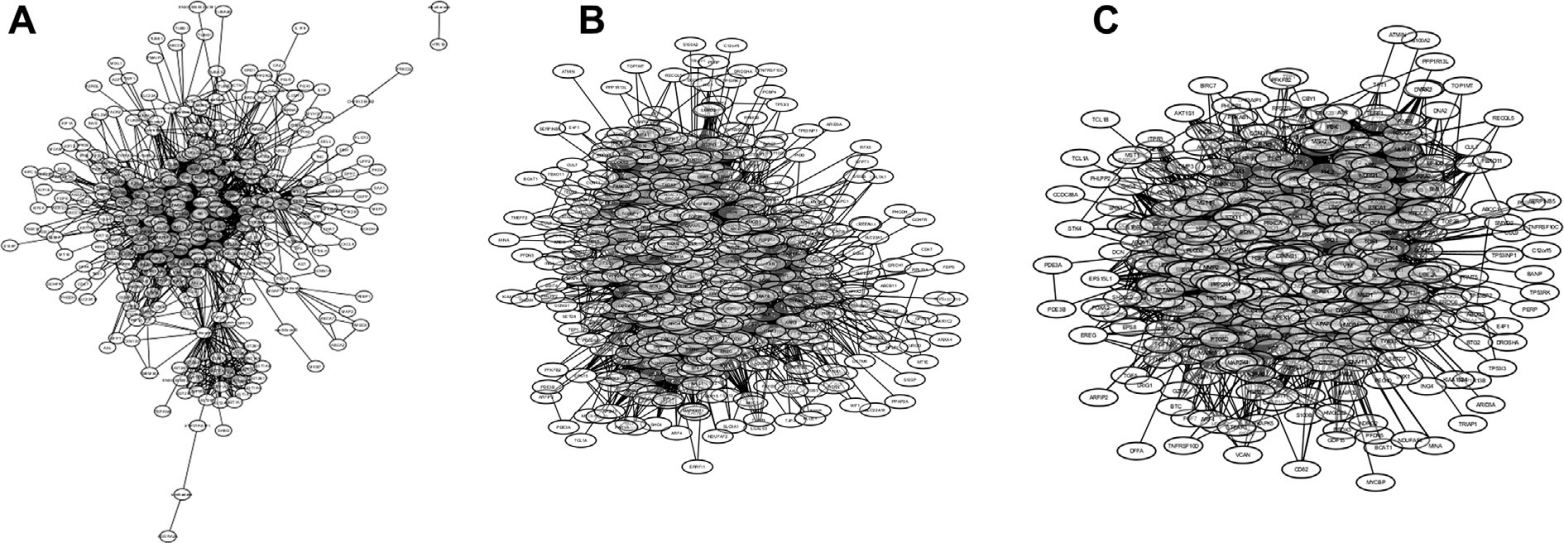
**MTA, DDA, and MTA–DDA human interactomes** **A**. Subnetworks of 2-methoxyestradiol, colchicine, combretastatin A4, docetaxel, epothilone, epothilone B, estramustine, nocodazole, noscapine, paclitaxel, podophyllotoxin, spongistatin, tasidotin, vinblastine, vincristine, vindesine, vinorelbine were merged to produce the integrated MTA interactome. **B.** The doxorubicin compound was used to produce the DDA interactome. **C.** The paclitaxel and doxorubicin subnetworks were merged to produce the human MTA–DDA interactome Subnetworks were constructed using STICH 4.0 database http://stitch.embl.de/ with the following criteria: a confidence score of 0.500 with 500 interactions; forcing search saturation; and all prediction methods being active. The MTA, DDA and MTA–DDA networks were created using Cytoscape version 3.4.0 [46] and merged using the Merge Networks plugin [http://www.cytoscape.org/plugins2.php]. MTA, microtubule-targeting agent; DDA, DNA-damaging agent.

To determine if MTA and DDA networks share common nodes, a DDA network analysis was performed. The obtained DDA network was then merged with the aforementioned MTA network. The doxorubicin subnetwork (DDA) presented 510 nodes and 6510 edges (Figure 3). The DDA network had a positive correlation for different topological features evaluated, for example, node degree (*R* = 0.634; *R*^2^ = 0.727), BC (*R* = 0.981; *R*^2^ = 0.825), and CC (*R* = 0.778; *R*^2^ = 0.584). These results suggest the presence of functionally-essential nodes within the network (topological axes) (Figure 3). The topological axes in the DDA network were obtained based on node degree. We found that *TP53*, *UBC*, *AKT1*, *MYC*, *EGFR*, *SRC*, *EP300*, *JUN*, *CREBBP*, and *CCND1* proteins were key components. These proteins regulate cellular processes such as apoptosis, cell differentiation, cell cycle control, replication, stress response, and transcriptional activation (Table 3).

The paclitaxel subnetwork was the principal hub of the MTA interactome with 166 nodes and 1497 edges and represents 33.8% of the MTAs network. The paclitaxel and doxorubicin subnetworks were merged to produce the MTA–DDA integrated network. The MTA–DDA network shows a positive correlation for all the topological parameters evaluated, including node degree (*R* = 0.734; *R*^2^ = 0.805), BC (*R* = 0.953; *R*^2^ = 0.821), and CC (*R* = 0.953; *R*^2^ = 0.821). In addition, the merged MTA–DDA network shared the same essential topological axes for the DDA network (Table S1). The functional profile of the MTA–DDA network revealed only one functional domain associated with the regulation of biological process, apoptosis, cellular cycle, and response to cellular stress.

## Discussion

The MTA integrated network was developed to describe the basal drug–protein interactions in a non-cancerous cell. The induction and regulation of apoptosis by MTAs should be considered as the central hub for all the subnetworks. However, a direct interaction between tubulin and any MTA was not observed, which opens two possibilities: (1) the apoptotic induction is mediated by secondary targets and (2) there is a common MTAs interactome core in the cell.

Our network analysis indicates that MTAs have common functional mechanisms, consistent with experimental observations showing that low concentrations of these compounds inhibit mitosis in a synergistic fashion [26]. The network analysis also allows the identification of interactions related to mitosis and microtubule activity during interphase.

It has been proposed that the functional advantages of MTAs are based on their inhibition of mitosis during interphase [27]. Our MTA interactome analysis revealed proteins and signaling pathways that are key points of regulation by these compounds, which include apoptosis, cell proliferation, cell cycle, and drug catabolism. However, these advantages of MTAs are primarily observed *in vitro* at the cell culture level [28], [29], as these compounds are associated with cytotoxicity *in vivo* [30], [31] and have harmful effects on rapidly dividing cells [32].

The cells of solid tumors show a high proliferation rate but a low apoptotic index [27], also known as the proliferation rhythm paradox [33]. The secondary effects of these compounds are observed mostly in fast-growing cells, such as haematopoietic and epithelial cells, leading to the conclusion that the main target of MTAs is the microtubules of interphase cells [27]. One of the main functions of interphase microtubules is to serve as “railways” for protein, vesicle and organelle trafficking [34]. The MTA interactome in this study revealed a large number of potential secondary targets (nodes), which were represented as client proteins of the interphase microtubules.

Two of these microtubule accessory proteins, dyneins DYNC1H1 and SYNC2H1 that transport cellular elements, were identified as topological axes for the MTA interactome. Taxanes antagonize the androgen receptor signaling pathway in prostate cancer cells by blocking dynein-mediated protein trafficking along interphase microtubules [35], [36], and paclitaxel decreases the endocytic trafficking of epidermal growth factor receptor in lung cancer cells [37]. In normal cells, these effects on dynein function lead to the accumulation of proteins in the cytosol and ultimately to apoptosis. Poruchynsky et al showed that in cells treated with doxorubicin, the proteins ATM, ATR, DNA-PK, RAD50, MRE11, p95/NBS1, p53, 53BP1, and p63, which are involved in DNA repair, were sequestered when vincristine and paclitaxel were added [20].

One of the most significant biological functions of the MTA interactome as represented by microtubule accessory proteins was the regulation of apoptosis. In the MTA interactome, both the catalytic α and the constitutive β subunits of chaperone HSP90 were revealed to be essential topological axes. HSP90 is vital in the folding process of several proteins involved in cell proliferation and apoptosis. Both subunits bind to the acetylated tubulin in a similar fashion as Akt and p53 [38], two other topological axes of the network. In fact, it is known that the cytosolic accumulation of p53 is induced by paclitaxel, vincristine, and nocodazole in lung cancer cells [39].

Furthermore, Pcl-2, which is known to interact with paclitaxel and initiates the pro-apoptosis cascade [40], was also identified as a topological axis for the MTA interactome. In human leukaemia cell lines and in the cells of patients with chronic lymphocyte leukaemia treated with paclitaxel, there is a significant increase in the expression of cytochrome c, caspase 3 and PARP, as well as a decreased JNK activity [41]. It is of note that the proteins JNK, caspase 3, and PARP were also identified as topological axes.

Another essential node that is tightly associated with apoptosis is the protein Bim_EL_ (MCL2L11), which is known as the most important physiological antagonist of survival proteins that are predominant in T lymphocytes. When phosphorylated, Bim_EL_ can leave the microtubule and be cleaved by caspase 3, thus activating mitochondrial- and/or receptor-based apoptotic mechanisms [42]. Our study is consistent with the observation that a cell can enter apoptosis through the interaction of caspase 3 and Bim_EL_ [42].

A second functional domain identified in the MTA interactome is related to xenobiotic metabolism. This type of metabolism consists of two phases, the first involving CYP450 proteins that detoxify or bioactivate chemical compounds through chemical functionalization and the second involving the exposure of molecules to conjugation reactions to make them more hydrophilic and susceptible to degradation [43]. The metabolism of paclitaxel is mediated by CYP1A2, 1B1, 2A6, 2C9, 2E1, and 3A4 [44], with 1A2 and 2C9 identified as the essential topological axes via the interactome analysis.

Using the non-cancerous cell MTA interactome, the protein components responsible for the induction and regulation of apoptosis both at the mitochondria and receptor levels could be revealed. Another functional component we find is the metabolic mechanisms for drug elimination, which was identified through several proteins involved in xenobiotic metabolism. These results suggest that the MTAs disrupt essential regulators of normal cell physiology present in both healthy and cancerous cells, mainly dependent on their effects on interphase microtubules.

## Materials and methods

### Protein–compound interaction network design

A list of agents targeting microtubules was obtained via a literature search, and the classification system developed by Bazan et al [45] was used as a reference. The names of the compounds were searched in the PubChem database to obtain the corresponding International Chemical Identifier (InChI) code. Interaction networks were created for each of the following compounds: (1) MTAs, including 2-methoxyestradiol, colchicine, combretastatin A4, docetaxel, epothilone, epothilone B, estramustine, nocodazole, noscapine, paclitaxel, podophyllotoxin, spongistatin, tasidotin, vinblastine, vincristine, vindesine, and vinorelbine; and (2) DDAs, including doxorubicin and darboplatin. The initial subnetworks for MTAs and DDA were obtained using STICH 4.0 database (http://stitch.embl.de/), with the following criteria: a confidence score of 0.500 with 500 interactions, forcing search saturation, and all prediction methods being active.

### Construction of merged MTA networks and topological analysis

The MTA subnetworks were imported into Cytoscape version 3.4.0 [46] and merged using the Merge Networks plugin [http://www.cytoscape.org/plugins2.php] included in Cytoscape by default. The following integrated network topology parameters were obtained using the NetworkAnalyzer plugin: level (k, degree), indicating how one node is connected with the others; intermediation (BC), indicating the number of shortest paths that pass through a node; CC, indicating which nodes are closer to the centre of the network; stress, indicating how many times a particular node is part of different shortest paths; and the clustering coefficient. As a control, random networks with 363 nodes and 2327 connections were generated with the mean *k* values of the integrated network using the Randomizer Network plugin [47].

The integrated network was analyzed using the MCODE plugin [48] to detect the main clusters, and GO analysis was performed for both the integrated network and the clusters using the Biological Network Gene Ontology (BiNGO) plugin. Hypergeometric distribution and false discovery rate (FDR) correction were adopted to determine the functional richness level for each network in each category. The FDR correction with a significance level of 0.05 is shown for two descriptive categories of the clusters, the first representing biological function and the second representing the node mapping in KEGG [49].

A network between paclitaxel and doxorubicin was merged and analyzed to test the hypothesis about a synergistic effect between MTAs and DDAs similarly as described above.

## Authors’ contributions

GM designed the project and AG performed all the bioinformatics analysis. AG and GM wrote the manuscript. Both authors read and approved the final manuscript.

## Competing interests

The authors have declared no competing interests.

## Supplementary material

Supplementary Table S1 Biological functions of MTA subnetworks and MTA–DDA interactome
